# Association between Genotype, Presentation, and Outcome in Childhood Idiopathic and Hereditary Pulmonary Arterial Hypertension

**DOI:** 10.3390/jcm11247331

**Published:** 2022-12-09

**Authors:** Zhuoyuan Xu, Hongsheng Zhang, Chen Zhang, Qiangqiang Li, Hong Gu

**Affiliations:** Paediatric Cardiology Department, Beijing Anzhen Hospital, Capital Medical University, No. 2 Anzhen Road, Chaoyang District, Beijing 100029, China

**Keywords:** paediatric, genetic, pulmonary arterial hypertension, pathogenic, outcome

## Abstract

Background: Paediatric-onset idiopathic/hereditary pulmonary arterial hypertension (IPAH/HPAH) is partially linked to genetic factors that may also affect treatment response and outcome. The relation between clinical characteristics and pathogenicity of gene variants in childhood IPAH/HPAH is still not well understood. Methods: We retrospectively analyzed IPAH/HPAH paediatric patients aged between 3 months and 18 years under follow-up at a large tertiary referral center. Whole-exome sequencing focused on PAH high-risk genes was performed in all patients. Pathogenicity grading of gene variant sites was assessed using ClinVar and population frequencies. The association between gene variants and death was studied using Cox proportional multivariate models. Results: Overall, 129 patients (54.3% females; 91.5% on PAH therapy) with a median age at diagnosis of 6.8 (IQR 3.4–10.7) years were included. A relevant PAH gene variant was detected in 95 patients (73.6%). The most common variants were in the BMPR2 (*n* = 43, 3%) gene. Over a median follow-up period of 27.6 months, 26 children died. The presence of a likely pathogenic genetic variant was significantly associated with survival (HR: 3.56, *p* = 0.005) on multivariable Cox analysis. The number of PAH-specific drugs at presentation was associated with better survival in the cohort with pathogenic variants (*p* = 0.02). Conclusions: Pathogenic/likely pathogenic genetic variants are prevalent in children with PAH and are related to a worse prognosis irrespective of other recognized risk factors in this population. Combination PAH therapy was associated with superior prognosis in children with pathogenic variants or BMPR2 variants. Therefore, proactive medical therapy should be employed in this population.

## 1. Introduction

Pulmonary arterial hypertension is a rare condition carrying a very poor prognosis [1]. Before the availability of disease-targeting drugs, the median survival time without treatment was 2.8 years for adult patients and less than one year for children [2]. The majority of cases in adults and children have a secondary cause. This includes, for example, left heart disease, lung disorders, liver disease and congenital heart disease. A relatively small proportion of adult patients are found to have idiopathic or hereditary pulmonary hypertension (IPAH/HPAH). These patients present earlier and have a worse prognosis than adults with other forms of PAH. It has also become clear that many cases of PAH presumed idiopathic may also have a genetic etiology, with rare genetic variants likely to contribute to around 11% of adult IPAH/HPAH. The diagnosis of heritable PAH (HPAH) is generally based on the presence of a positive family history and/or a confirmed causative genetic mutation [3]. In contrast, idiopathic PAH (IPAH) is usually assumed if no underlying disease known to be associated with PAH is present [3]. As a rising number of genes that may be associated with PAH are discovered, there is increasing understanding of an overlap between IPAH and HPAH. In fact, 10–20% [4] of sporadic IPAH cases have been found to have a disease-causing genetic component. Much of our understanding of paediatric-onset PAH comes from extrapolation from adult data. Currently, there are relatively few case series describing paediatric cohorts. This is in part because of the condition’s rarity, with a reported prevalence of just 4.8–8.1 cases per million [5]. There are also important differences between adult-onset and paediatric-onset PAH, including sex balance, disease aetiology, clinical presentation, and response to therapy. In adults, an increased prevalence of IPAH/HPAH is observed in females, while in children the sex distribution is more balanced [5]. Etiologically, paediatric-onset PAH has a higher proportion of IPAH/HPAH and has heavier genetic burden than adult-onset IPAH/HPAH, with rare genetic factors contributing to approximately 35% of paediatric IPAH/HPAH cases [5].

The importance of genetic causes of paediatric IPAH/HPAH is highlighted in a recent paediatric consensus statement. This document also emphasises implications for therapy in variant carriers and provides more information for family counselling/screening and follow up for asymptomatic family members with variants [6]. However, genetic aetiology has not been generally included as a routine factor for the proposed risk-stratification criteria to guide therapy in children with PAH [3]. Increasing access to gene sequencing and increased accuracy of variant interpretation has enabled a more precise understanding of the potential role of genetic variation in the aetiology of PAH, in both adult and paediatric populations. Rare pathogenic mutations in BMPR2 contribute to paediatric-onset IPAH/HPAH with a frequency similar to that of adult-onset disease. However, rare pathogenic variants in TBX4 are more common in paediatric patients compared with adults, explaining around 8% of paediatric IPAH based on a large US cohort [5]. De novo variants and rare pathogenic variants in BMPR2, TBX4, and SOX17 currently explain most of the known genetic burden in paediatric PAH in western countries [7]. In Asian cohorts, pathogenic variants in ACVRL1 and GDF2 (also known as BMP9) may, however, play a more prominent role [8,9]. Particularly in the paediatric population, where genetic aetiology is more common, reaching a genetic diagnosis is of great importance. Genotype may also influence clinical course and prognosis [1,3] and may contribute to family screening and family counselling. There is some evidence that specific targeted therapy may be more effective in patients with pathogenic variants in particular genes [3].

Data on large cohorts based on Asian paediatric-onset PAH to identify the specific mix of genetic causes of PAH, as well as the natural history and response to therapy, are still lacking. We aimed to describe the association of genotype with clinical characteristics and prognosis based on the biggest Asian paediatric IPAH/HPAH cohort with whole-exome sequencing (WES) where all patients were followed at a large tertiary paediatric PAH centre with standardized PAH therapy protocols. In addition to the definitive PAH-causative genes, the study also provides phenotype information on newly identified PAH-related genes.

## 2. Methods

### 2.1. Study Population

This was a retrospective analysis of paediatric patients from 21 provinces across China with age at PAH onset between 3 months and 18 years with unexplained PAH who were referred to the Beijing Anzhen Hospital between March 2008 and October 2021.

We assessed each patient by clinical history, physical examination, current therapy, WHO cardiac function classification (WHO FC), 6-min walking distance (6MWD) (for children over 6 years of age who were able to comply with the instructions), and blood testing. All patients underwent gene sequencing as described below. At diagnosis, 86 patients (66.7%) underwent cardiac catheterization, and 43 clinically unstable patients (33.3%) initially underwent only a noninvasive echocardiographic investigation confirming the diagnosis of PAH [10]. Pulmonary arterial hypertension was diagnosed based on the relevant guideline recommendation at the time of diagnosis in all patients [10,11,12]. Therefore, a mean PAP ≥ 25 mmHg (mPAP), pulmonary artery wedge pressure (PCWP) ≤ 15 mmHg, and PVR > 3 Wood units measured by right heart catheterization (RHC) were used as diagnostic criteria. Acute vasodilator testing (AVT) was performed with inhaled Iloprost during cardiac catheterization in patients with a mean PAP over 40 mmHg at baseline, and a positive response was defined according to current guidelines as a decrease in mPAP of at least 10 mmHg to <40 mmHg, with a stable cardiac output [13]. Patients with any secondary aetiologies of pulmonary hypertension as suggested by ECG, chest X-ray, echocardiogram, abdominal ultrasound, pulmonary CT, laboratory tests, lung function test, and right heart catheterization, such as left heart disease, lung diseases, liver diseases, connective tissue diseases, and drug/toxin-induced PAH, as well as persistent PH of the newborn syndrome, were excluded. Risk stratification was based on baseline parameters, following the published criteria of the World Symposium on Pulmonary Hypertension [11].

After the initial assessment, the patients were followed up at approximately 3- to 6-month intervals. Treatment of patients was performed in accordance with relevant guidelines [3,11]. The primary endpoints of the study were all-cause mortality. As no patient underwent lung transplant, this was not considered part of the study endpoint.

### 2.2. Genetic Testing and Whole-Exome Data Analysis

Whole-exome sequencing (WES) was performed by BestNovo (Beijing, China) Medical Laboratory to detect variants in PAH-related genes. To this end, genomic DNA was extracted from peripheral venous blood using a DNA Isolation Kit (Roche, Indianapolis, IN, USA). The GenCap enrichment kit (MyGenostics, Beijing, China) was used to capture exons of candidate genes. HiSeq 2500 (Illumina Inc., San Diego, CA, USA) was sequenced with an average effective sequencing depth of 100 bp, and bioinformatics analysis was performed. The pathogenicity of the mutations was estimated using predictive software (Polyphen-2, SIFT, and PANTHER), and variants were confirmed by Sanger sequencing. According to the previous studies, expert review, and guidelines [6,14,15,16,17,18,19], we included BMPR2, ACVRL1, TBX4, NOTCH3, KCNK3, ABCA3, GDF2, THBS1 (also known as TSP1), HTR2B, SMAD9, ENG, TOPBP1, SMAD1, KCNA5, CPS1, CAV1, FBN1, BMPR1B, SOX17and biallelic mutations in EIF2AK4 as high-risk genes with PAH in our study. Variants were classified as pathogenic/likely pathogenic (P/LP) if they have been reported as pathogenic or likely pathogenic in ClinVar (https://www.ncbi.nlm.nih.gov/clinvar/; accessed on 2 April 2022) or reached pathogenic/likely pathogenic classification using the ACMG criteria (on date April 2022). Other variants were classified as variant of uncertain significance “VUS” if they had a frequency in The Genome Aggregation Database (gnomAD v2.1.1; https://gnomad.broadinstitute.org/help (accessed on 25 October 2022)) of <1 in 1000. This is a permissive cutoff, chosen because of the low reported penetrance of some disease-causing variants in BMPR2 [20]. Variants with higher population frequencies were classified as benign.

### 2.3. Statistical Analysis

All analyses were performed using R Version 4.2.0 (R Foundation, Vienna, Austria). Continuous variables are presented as mean ± standard deviation or median (interquartile range) and categorical variables as number and percentages. Differences between groups were assessed using Wilcoxon for continuous variables and chi-square test for categorical variables. A two-sided *p*-value < 0.05 was considered indicative of statistical significance. Kaplan–Meier survival curves were used to describe differences between groups in terms of calendar age as in paediatric cohort, as well as survival from diagnosis to death or lung transplantation, with comparisons made using the log-rank test. Univariate and multivariate Cox regression analyses were used to identify variables predictive of survival. This observation was robust to alterations in the model variables. A stepwise analysis (StepAIC) was used to select a simple model with the most predictive variables based on the Akaike information criterion. The 6MWD and WHO class were not used due to the difficulty in assessing these in the paediatric population and therefore missing data in the younger children.

## 3. Results

### 3.1. Demographics and Biometrics

A total of 129 paediatric patients were included in this analysis. The baseline clinical characteristics are summarized in Table 1. The median age at diagnosis of the patients was 6.8 (IQR 3.4 to 10.7). Female sex predominance was observed in the cohort (*n* = 70, 54.3%).

Dyspnea and reduced exercise capacity were the most common presenting reasons and were present in 48 patients (37.2%). Syncope was also a frequent presentation, found in 36 patients (27.9%), with 24 children also having a history of recurrent syncope. Epistaxis was described in three patients and hemoptysis in four patients. Time from onset of symptoms to diagnosis was 6.0 (IQR 1.0 to 24.0) months. At the time of diagnosis, the proportion of patients with WHO functional class I, II, III, and IV was 3%, 43%, 35% and 19%, respectively. The median baseline BNP level was elevated at 261.0 (54.8, 758.5) pg/mL.

All of 129 patients underwent echocardiography scan using the peak tricuspid regurgitation velocity (TRV) at baseline as the key variable for assigning the echocardiographic probability of PAH. In our cohort, mean TRV was 446.5 ± 79.7 cm/s. Other echocardiographic findings of PAH, estimating pressure and signs of RV overload and/or dysfunction, including reduced mean tricuspid annular plane systolic excursion (TAPSE), increased right to left atrial area ratio (RA/LA area ratio) and increased basal RV/LV ratio, are summarized in Table 1. Increased systolic to diastolic (SD) duration ratio reflected decreased global RV performance in our cohort.

Eighty-six of one hundred twenty-nine patients underwent baseline RHC. Of these, 73 patients were subjected to acute vasodilator response testing (AVT), 9 of whom were responders.

Twenty-nine patients (22.5%) had small shunts including atrial septal defect (ASD), ventricle septal defect (VSD) and patent ductus arteriosus (PDA), which were all considered coincidental rather than causal for PAH.

**Figure 1:**
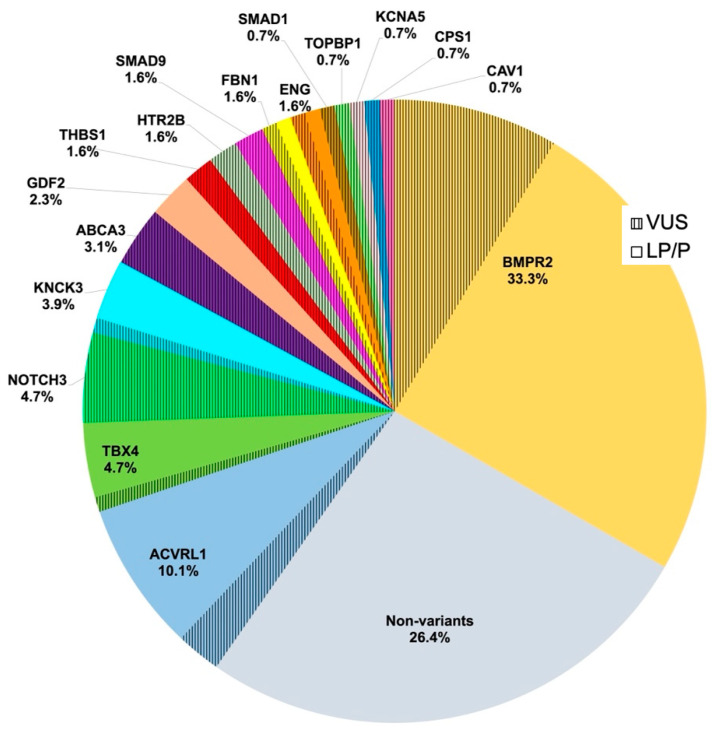
Relative contributions of 17 established PAH risk genes detected in idiopathic paediatric PAH in a cohort of 129 cases. Risk genes included BMPR2, ACVRL1, TBX4, NOTCH3, KCNK3, ABCA3, GDF2, THBS1 (also known as TSP1), HTR2B, SMAD9, ENG, TOPBP1, SMAD1, KCNA5, CPS1, CAV1, and FBN1. PAH, pulmonary arterial hypertension; VUS, variants of uncertain (or unknown) significance; LP/P, likely pathogenic or pathogenic variants in PAH-related genes; % is the percentage of both LP/P and VUS in each gene.

Ninety-five patients (73.6%) had pathogenic variants in PAH panel genes. The distribution of genotypes and variant statuses is shown in Figure 1. As depicted, the most commonly identified variants were in BMPR2 (*n* = 43, 33%). The next most common genes with variants were ACVRL1 (*n* = 13, 10%), TBX4, and NOTCH3 (6 patients each, 5%), although the latter group of variants was classified as VUS. De novo variants were found in 16 patients (12.4%): 9 in BMPR2, 3 in TBX4, 2 in ACVRL1, 1 in KCNK3 and 1 in GDF2. Only 5 patients (3.9%) had a familial history of PAH.

### 3.2. Associations between Genotype and Clinical Characteristics

Clinical characteristics in patients with or without likely pathogenic variants (or VUSs in BMPR2) are shown in Supplementary Figure S1. Patients with BMPR2 or any other pathogenic variants had older median diagnostic age (107.1 [50.7, 144.7] vs. 67.2 [33.1, 105.4] months, *p* = 0.0033) and worse hemodynamic characteristics, with lower cardiac index (CI) (3.0 [2.5, 4.1] vs. 3.5 [2.9, 4.5] L/min/m^2^, *p* = 0.041) and raised indexed pulmonary vascular resistance (PVRI) (20.4 [11.0, 28.2] vs. 15.1 [5.8, 22.5] WU·m^2^, *p* = 0.032). No significant statistical differences were seen in WHO FC, BNP and RAP. Notably, as shown in Supplementary Figure S1a, no pathogenic variants in known PAH genes were found in any of the patients whose age of diagnosis was less than 7.5 months.

### 3.3. Medical Treatments

In the overall cohort, most patients (91.5%, *n* = 118) received initial targeted PAH therapy, including four patients with calcium channel inhibitor (CCB) treatment. The targeted therapy included PDE-5 inhibitors (57%, *n* = 74), endothelin receptor antagonists (ERA) (82.9%, *n* = 107), Treprostinil infusion (17.8%, *n* = 23), and oral prostacyclin analogon (7.8%, *n* = 10). Among the patients, 34.1% (44/129), 41.1% (53/129), and 16.3% (21/129) received initial monotherapy, double combination therapy, and triple therapy, respectively. Therapy for heart failure included diuretics (30%, *n* = 39) and inotropic agents (30%, *n* = 39).

### 3.4. Outcomes

During a median follow-up of 27.6 [IQR 12.0 to 43.2] months, 26 patients (22.5%) died, with 18 (18/26, 69.2%) of them being BMPR2 mutation carriers, 4 (4/26, 15.4%) ACVRL1 mutation carriers, one a KCNK3 mutation carrier, one an FBN1 mutation carrier, one an ENG mutation carrier, and one having no known genetic alteration. Eleven patients were included on the transplantation list, but no patient underwent heart–lung transplantation in our cohort. The median time from diagnosis to death was 18.8 (IQR 4.1 to 30.9) months. Among these patients, the diagnostic age was 96.5 ± 45.0 months, and age at death was 127.1 ± 64.9 months. Among patients with family histories, two (both BMPR2 mutation carriers) died at the age of 57.2 months and 160.3 months, respectively, and three patients (two are ACVRL1 mutation carriers and one is a KCNK3 mutation carrier) are on the waiting list for transplantation. The overall endpoint of survival, estimated by Kaplan–Meier analysis, at 1, 3, and 5 years after diagnosis was 94.2%, 78.8%, and 75.9%, respectively.

### 3.5. Variables Related to Paediatric IPAH Mortality

**Figure 2:**
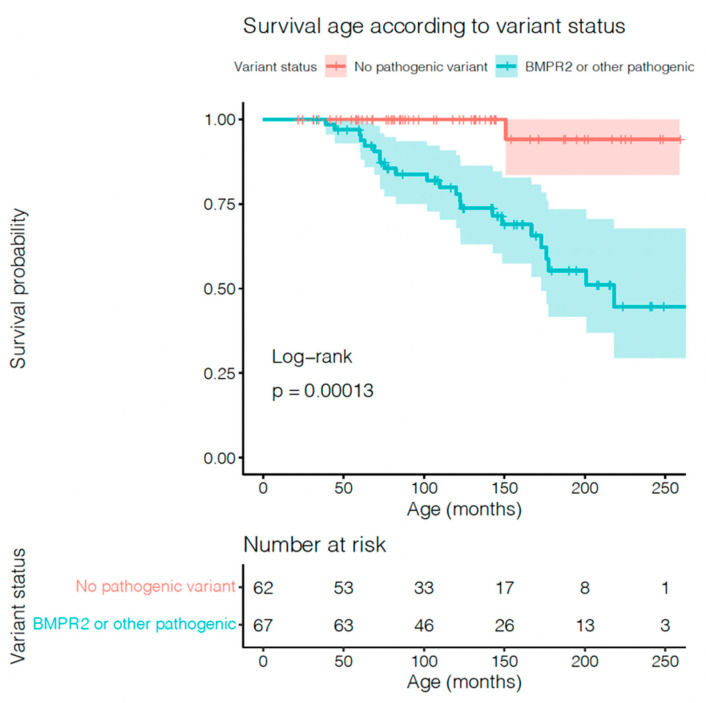
Relationship between genetic variant status and paediatric IPAH/HPAH mortality by Kaplan–Meier survival analysis in patients with BMPR2 (pathogenic and VUS) or other genetic pathogenic variants, and without variants.

Patients with any pathogenic variants had a worse prognosis compared with patients with non-pathogenic variants, as shown in Figure 2 (*p* = 0.0001). No significant statistical difference was found in patients with VUS BMPR2 compared with pathogenic BMPR2 (see Supplementary Figure S2). The final multivariate model included sex (male), BNP level, PVRI, mPAP/mSAP, AVT-positive responders, as well as patients with BMPR2 or other pathogenic variants. Results of univariate and multivariate analysis of full model and final model are shown in Table 2 and Table 3. In multivariate Cox regression analysis, including available variables reported to define low- or high-risk patients with paediatric PAH, the presence of a likely pathogenic genetic variant remained significantly associated with worse survival (HR: 3.563, *p* = 0.005). Adding the number of PAH medications to this model did not significantly improve the fit, and the number of medications was not significantly associated with survival in the overall cohort (*p* = 0.23). However, the number of medications was significantly associated with survival in the cohort with pathogenic variants (*p* = 0.008). Notably, none of the patients with BMPR2 variants under triple therapy died, but those who did not receive the treatment had a significantly worse prognosis (*p* = 0.019).

## 4. Discussion

We reviewed the genotype and phenotype of paediatric patients with HPAH/IPAH at a large tertiary referral centre. The size of the study (*n* = 129), number of referral regions (*n* = 21), and standardized treatment strategies should allow generalization of the findings to the overall clinical Chinese practice. The median age at diagnosis of our cohort was similar to that of the Registry to Evaluate Early And Long-term pulmonary arterial hypertension disease management (REVEAL Registry) [21] (6.8 vs. 7 years), older than that of the UK cohort [22] (6.8 vs. 4.3 years), but younger than that of the Japan study [23,24] (6.8 vs. 8.0–8.5 years). The 5-year survival from diagnosis for our cohort was 76%, and therefore similar to the previous studies [21,23] (74% in REVEAL Registry, 76% in Japanese cohort).

Genetic factors likely play an important role in the mechanism of IPAH and in severity of the disease in childhood populations, as we could confirm that 74% of the variants were present in our HPAH/IPAH cohort. In the current study, 23% of patients reached the primary endpoint of death, with the worst outcomes found in BMPR2 variants. The association between genetic alterations and all-cause mortality was confirmed on multivariate Cox proportional analysis after adjusting for other recognized risk factors. Patients with any rare missense variant in BMPR2 appeared to have similar hemodynamic and survival characteristics as those with other confirmed pathogenic variants. This suggests that even not classically Mendelian disease-causing variants might affect disease expression and prognosis in paediatric PAH. Amongst other genes, patients with VUS had survival and hemodynamic profiles similar to those of patients without identified rare variants in known PAH genes, indicating that this cohort may be considered lower risk. The presence of a likely pathogenic or disease-modifying variant, in contrast, was associated with a significantly worse prognosis in this patient population, even when hemodynamics was considered. As we presented in the Results section, we included variables suggested in the 2019 EPPVDN Risk Score Sheet for Paediatric pulmonary hypertension. The presence of a pathogenic or likely pathogenic genetic variant consistently maintained a stable and significant association with prognosis, even in the presence of other recommended factors. The importance of genetic diagnosis in paediatric unexplained PAH should be highlighted, and genotype might be more accurate in predicting long-term survival than the other factors.

In examining the effect of treatment in the whole cohort or in those with any pathogenic variant, survival does appear to be significantly associated with the number of medications, although there is also a signal for significance in the pathogenic variant group. It has to be noted that the number of patients receiving no medications is very small, and these are likely to be a selected cohort, perhaps with comorbidities or clinical features that made them at presentation unsuitable for treatment. However, if we take an isolated look at those with BMPR2 variants—a group previously reported to respond well to PGI2 inhibitors in particular [23]—it becomes apparent that there were no deaths in the patients receiving triple PAH medications. There appears to be a significant survival benefit associated with increasing number of medications in patients with BMPR2 variants. This suggests that patients with any pathogenic variant can benefit from initiation of combination therapy. Perhaps the presence of a pathogenic or likely pathogenic genetic variant can be part of paediatric-specific risk stratification in order to raise awareness of IPAH risk and progression to trigger further treatments.

In our study, patients with BMPR2 or other pathogenic variants had a higher age at diagnosis compared with patients without variants. This is consistent with the US paediatric IPAH cohort, with the most common age of onset at 10 years in BMPR2 variants, which is older than TBX4 mutation carriers, whose age of onset was less than 5 years [25]. In contrast, adult-onset patients with BMPR2 variants were much younger than those with other variants [4,26]. That may indicate that patients with early onset may not necessarily have a poor prognosis.

The genetic background of IPAH is complex, and the phenotype is highly heterogeneous. In our study, we only included patients older than 3 months, which by definition excluded those with pulmonary hypertension of the newborn (PPHN). Of note, our patients whose age of onset was less than 7.5 months were all non-variant patients, indicating that there might be gene backgrounds that we have not found in these infant-onset patients, or they might be affected by other unknown factors.

Right heart function is an important factor in determining the prognosis of patients with PAH [27]. Recent research showed an effect of BMPR2 mutation specifically on the right ventricle, which compromised intrinsic function, even in nonoverloaded states, suggesting a deleterious effect of this mutation in right ventricular adaptation in carriers developing the disease and a compromised response of these patients to standard therapy and lung transplantation [28]. There are an increasing number of targeted therapy studies for BMPR2 gene defects. Although these data are not yet available for children, it is increasingly clear that individualized treatment for patients with specific genetic defects, especially BMPR2, could be of great benefit.

However, the contribution of individual genes in PAH is likely heterogeneous across different genetic ancestries. Previous data suggest that ACVRL1 variants may exist with higher frequency and worse outcomes in Asian paediatric cohorts, [23] and this is consistent with our data. GDF2 (also known as BMP9) was identified as a significant gene in the Chinese adult IPAH cohort and the second in frequency to BMPR2. De novo rare variants in our cohort were not as common as in the US cohort (15% vs. 13%) [5]. Clearly, the results of genetic studies predominantly in individuals of European ancestry may not be generalizable to all other populations. Our study—as one of the larger paediatric studies—gives some evidence to the difference in genetic ancestry, but larger studies of children with greater diversity are needed to define ancestral-specific genetic factors and their overall role in paediatric-onset PAH.

### Strengths and Limitations

To the best of our knowledge, the current report represents one of the most detailed studies on the classification of genetic pathogenicity grades in China. The study comprehensively evaluates the prevalence of HPAH/IPAH, its phenotype and its associated risk factors in the different subgroups of genotype based on the largest paediatric PAH diagnosis and treatment center in mainland China with relatively uniform and standardized treatments. The current study definition of PAH is in line with the guidelines at the time of the study period (mPAP > 25 mmHg). Since then, the new guideline documents have changed the definition to a lower mPAP of 20 mmHg. However, as most patients with severe PAH present with much higher pulmonary pressures, this should not relevantly affect the results of the study. Our study was a single-center study, resulting in possible measurement of confounders that vary between centers. A prospective registration system of Chinese paediatric IPAH should be established to clarify prevalence, mortality, risk factors, and especially drug efficacy.

## 5. Conclusions

The presence of a pathogenic or likely pathogenic genetic variant was consistently associated with worse prognosis, even in the presence of other relative factors. Despite significant advances in management, mortality remains significant in patients with HPAH/IPAH, especially those with specific gene variants. Initial combination therapy might improve short- to mid-term survival, even with BMPR2 pathogenic variants to some extent. Therefore, undertaking genetic sequencing as a screening method can be recommended to support risk stratification and guide individualized combination PAH targeted therapy.

## Figures and Tables

**Table 1:** Baseline clinical characteristics of patients with idiopathic pulmonary arterial hypertension and in subgroups.

	Overall (N = 129)
Diagnostic age, months	81.3 (40.8, 128.2)
WHO III-IV, *n* (%)	70, 54.3%
6MWD available, *n*	45, 34.9%
6MWD, m	427.7 ± 95.9
Time from onset of the symptom to diagnosis, months	6.0 (1.0, 24.0)
***Symptoms***	
Decline in activity, *n* (%)	48 (37.2)
Syncope, *n* (%)	36 (27.9)
Repeated syncope, *n* (%)	24 (18.6)
***Biomarkers***	
Hemoglobin, g/L	136.7 ± 21.6
HCT, %	41.0 ± 10.5
BNP, pg/mL	261.0 (54.8, 758.5)
ALT, U/L	21.0 (13.0, 31.0)
***Echocardiography***	
Peak tricuspid regurgitation velocity, cm/s	446.5 ± 79.7
TAPSE, mm	14.8 ± 4.2
S/D ratio	2.2 ± 1.0
RA/LA area	2.1 ± 1.0
Basal RV/LV ratio	1.2 ± 0.7
***Hemodynamics***	
RHC, *n* (%)	86, 66.7%
Height, cm	129.6 ± 27.7
Weight, kg	31.5 ± 17.9
mPAP, mmHg	67.6 ± 22.4
PVR, WU	17.9 (9.3, 27.2)
PVRI, WU·m^2^	18.5 (9.1, 27.2)
Cardiac index, L/min/m^2^	3.3 (2.6, 4.3)
Rp/Rs	1.0 (0.6, 1.3)
mPAP/mSAP	0.88 ± 0.32
AVT, positive/*n* (%)	9/83, 10.8%
AVT-PAMP, mmHg	62.6 ± 21.1
AVT-PVR, WU	13.8 (7.7, 20.0)
AVT-PVRI, WU·m^2^	14.3 (7.4, 22.7)
AVT-mPAP/mSAP	0.80 ± 0.30
***Risk stratification***	
lower risk	66
higher risk	63

Values are *n* (%), median (interquartile range), or mean (±standard deviation). N, number; WHO FC, World Health Organization functional class; 6MWD, 6-min walk distance; HCT, hematocrit; BNP, B-type brain natriuretic peptide; ALT, alanine transaminase; TAPSE, tricuspid annular plane systolic excursion; S/D ratio, systolic to diastolic duration ratio; RA/LA area ratio, right to left atrial area ratio measured in 4 chamber view; basal RV/LV ratio, basal right ventricle to left ventricle ratio measured in 4 chamber view; RHC, right heart catheterization; SvO_2_, mixed venous oxygen saturation; RAP, right atrial pressure; mPAP, mean pulmonary artery pressure; PVR, pulmonary vascular resistance; PVRI, indexed pulmonary vascular resistance; Rp/Rs, pulmonary vascular resistance/systemic vascular resistance; mPAP/mSAP, mean pulmonary artery pressure/mean systemic artery pressure; AVT, acute vasodilator testing.

**Table 2:** Univariate Cox regression model for IPAH/HPAH risk of mortality.

	Hazard Ratio (95% Confidence Interval)	*p* Value
Sex Male	2.08 (0.90–4.76)	0.085
BNP, pg/mL	1.00 (1.00–1.00)	0.001
PVRI, WU·m^2^	1.03 (0.98–1.08)	0.247
mPAP/mSAP	12.54 (2.07–76.08)	0.006
AVT positive	0.28 (0.06–1.21)	0.088
BMPR2 and pathogenic others	17.25 (2.33–127.88)	0.005
Diagnostic age (months)	0.99 (0.98–0.99)	<0.001
Rp/Rs	3.50 (1.28–9.54)	0.015
CI, L/(min·m^2^)	0.94 (0.58–1.52)	0.788
No medication	0.78 (0.49–1.27)	0.319
RAP (mmHg)	1.16 (1.01–1.34)	0.031
WHO class	1.34 (0.82–2.19)	0.245
6MWD, m	1.00 (0.99–1.01)	0.857

BNP, B-type brain natriuretic peptide; PVRI, indexed pulmonary vascular resistance; mPAP/mSAP, mean pulmonary artery pressure/mean systemic artery pressure; AVT, acute vasodilator testing; Rp/Rs, pulmonary vascular resistance/systemic vascular resistance; CI, cardiac index; RAP, right atrial pressure; WHO class, World Health Organization functional class; 6MWD, six-minute walk distance.

**Table 3:** Multivariate Cox regression model for IPAH/HPAH risk of mortality final model.

	Hazard Ratio (95% Confidence Interval)	*p* Value
Sex Male	4.70 (1.10–20.12)	0.037
BNP, pg/mL	1.00 (1.00–1.00)	0.018
PVRI, WU·m^2^	0.91 (0.83–0.99)	0.033
mPAP/mSAP	649 (22–18998)	<0.001
AVT positive	0.14 (0.02–0.89)	0.038
BMPR2 and pathogenic others	35.28 (2.95–421.73)	0.005

BNP, B-type brain natriuretic peptide; PVRI, indexed pulmonary vascular resistance; mPAP/mSAP, mean pulmonary artery pressure/mean systemic artery pressure; AVT, acute vasodilator testing.

## Data Availability

The data presented in this study are available on request from the corresponding author. The data are not publicly available due to data confidentiality reasons.

## Supplementary Materials

The following supporting information can be downloaded at: https://www.mdpi.com/article/10.3390/jcm11247331/s1, Figure S1: Clinical characteristics and hemodynamics in subgroups: Comparisons between patients with pathogenic and VUS in BMPR2 variants or pathogenic/likely pathogenic variants in other PAH related genes, and no pathogenic variants in age at diagnosis, WHO FC, BNP level, RAP, PVRI and cardiac index (CI). (*n* = 62 in no variants, *n* = 67 in BMPR2 or pathogenic/likely pathogenic variants in other PAH related genes); Figure S2: Kaplan-Meier survival analysis in patients with VUS BMPR2 and pathogenic BMPR2.

## References

[1] Humbert M., Kovacs G., Hoeper M.M., Badagliacca R., Berger R.M.F., Brida M., Carlsen J., Coats A.J.S., Escribano-Subias P., Ferrari P. (2022). 2022 ESC/ERS Guidelines for the diagnosis and treatment of pulmonary hypertension. Eur. Heart J..

[2] Benza R.L., Miller D.P., Barst R.J., Badesch D.B., Frost A.E., McGoon M.D. (2012). An evaluation of long-term survival from time of diagnosis in pulmonary arterial hypertension from the REVEAL Registry. Chest.

[3] Rosenzweig E.B., Abman S.H., Adatia I., Beghetti M., Bonnet D., Haworth S., Ivy D.D., Berger R.M.F. (2019). Paediatric pulmonary arterial hypertension: Updates on definition, classification, diagnostics and management. Eur. Respir. J..

[4] Evans J.D., Girerd B., Montani D., Wang X.J., Galie N., Austin E.D., Elliott G., Asano K., Grunig E., Yan Y. (2016). BMPR2 mutations and survival in pulmonary arterial hypertension: An individual participant data meta-analysis. Lancet Respir. Med..

[5] Welch C.L., Austin E.D., Chung W.K. (2021). Genes that drive the pathobiology of pediatric pulmonary arterial hypertension. Pediatr. Pulmonol..

[6] Morrell N.W., Aldred M.A., Chung W.K., Elliott C.G., Nichols W.C., Soubrier F., Trembath R.C., Loyd J.E. (2019). Genetics and genomics of pulmonary arterial hypertension. Eur. Respir. J..

[7] Welch C.L., Chung W.K. (2020). Genetics and Other Omics in Pediatric Pulmonary Arterial Hypertension. Chest.

[8] Lyu Z.C., Wang L., Lin J.H., Li S.Q., Wu D.C., Lian T.Y., Liu S.F., Ye J., Jiang X., Wang X.J. (2020). The features of rare pathogenic BMPR2 variants in pulmonary arterial hypertension: Comparison between patients and reference population. Int. J. Cardiol..

[9] Wang X.J., Lian T.Y., Jiang X., Liu S.F., Li S.Q., Jiang R., Wu W.H., Ye J., Cheng C.Y., Du Y. (2019). Germline BMP9 mutation causes idiopathic pulmonary arterial hypertension. Eur. Respir. J..

[10] Galie N., Humbert M., Vachiery J.L., Gibbs S., Lang I., Torbicki A., Simonneau G., Peacock A., Vonk Noordegraaf A., Beghetti M. (2015). 2015 ESC/ERS Guidelines for the diagnosis and treatment of pulmonary hypertension: The Joint Task Force for the Diagnosis and Treatment of Pulmonary Hypertension of the European Society of Cardiology (ESC) and the European Respiratory Society (ERS): Endorsed by: Association for European Paediatric and Congenital Cardiology (AEPC), International Society for Heart and Lung Transplantation (ISHLT). Eur. Respir. J..

[11] Hansmann G., Koestenberger M., Alastalo T.P., Apitz C., Austin E.D., Bonnet D., Budts W., D’Alto M., Gatzoulis M.A., Hasan B.S. (2019). 2019 updated consensus statement on the diagnosis and treatment of pediatric pulmonary hypertension: The European Pediatric Pulmonary Vascular Disease Network (EPPVDN), endorsed by AEPC, ESPR and ISHLT. J. Heart Lung Transplant..

[12] Abman S.H., Hansmann G., Archer S.L., Ivy D.D., Adatia I., Chung W.K., Hanna B.D., Rosenzweig E.B., Raj J.U., Cornfield D. (2015). Pediatric Pulmonary Hypertension: Guidelines From the American Heart Association and American Thoracic Society. Circulation.

[13] Sitbon O., Humbert M., Jais X., Ioos V., Hamid A.M., Provencher S., Garcia G., Parent F., Herve P., Simonneau G. (2005). Long-term response to calcium channel blockers in idiopathic pulmonary arterial hypertension. Circulation.

[14] Pattathu J., Gorenflo M., Hilgendorff A., Koskenvuo J.W., Apitz C., Hansmann G., Alastalo T.P. (2016). Genetic testing and blood biomarkers in paediatric pulmonary hypertension. Expert consensus statement on the diagnosis and treatment of paediatric pulmonary hypertension. The European Paediatric Pulmonary Vascular Disease Network, endorsed by ISHLT and DGPK. Heart.

[15] Liang K.W., Chang S.K., Chen Y.W., Lin W.W., Tsai W.J., Wang K.Y. (2022). Whole Exome Sequencing of Patients With Heritable and Idiopathic Pulmonary Arterial Hypertension in Central Taiwan. Front. Cardiovasc. Med..

[16] Kumar R., Mickael C., Kassa B., Sanders L., Hernandez-Saavedra D., Koyanagi D.E., Kumar S., Pugliese S.C., Thomas S., McClendon J. (2020). Interstitial macrophage-derived thrombospondin-1 contributes to hypoxia-induced pulmonary hypertension. Cardiovasc. Res..

[17] Delaney C., Sherlock L., Fisher S., Maltzahn J., Wright C., Nozik-Grayck E. (2018). Serotonin 2A receptor inhibition protects against the development of pulmonary hypertension and pulmonary vascular remodeling in neonatal mice. Am. J. Physiol. Lung Cell. Mol. Physiol..

[18] Cinelli E., Iovino L., Bongianni F., Pantaleo T., Mutolo D. (2016). GABAA- and glycine-mediated inhibitory modulation of the cough reflex in the caudal nucleus tractus solitarii of the rabbit. Am. J. Physiol. Lung Cell. Mol. Physiol..

[19] Liu X., Mei M., Chen X., Lu Y., Dong X., Hu L., Hu X., Cheng G., Cao Y., Yang L. (2019). Identification of genetic factors underlying persistent pulmonary hypertension of newborns in a cohort of Chinese neonates. Respir. Res..

[20] White R.J., Morrell N.W. (2012). Understanding the low penetrance of bone morphogenetic protein receptor 2 gene mutations: Another needle in the haystack. Circulation.

[21] Barst R.J., McGoon M.D., Elliott C.G., Foreman A.J., Miller D.P., Ivy D.D. (2012). Survival in childhood pulmonary arterial hypertension: Insights from the registry to evaluate early and long-term pulmonary arterial hypertension disease management. Circulation.

[22] Moledina S., Hislop A.A., Foster H., Schulze-Neick I., Haworth S.G. (2010). Childhood idiopathic pulmonary arterial hypertension: A national cohort study. Heart.

[23] Chida A., Shintani M., Yagi H., Fujiwara M., Kojima Y., Sato H., Imamura S., Yokozawa M., Onodera N., Horigome H. (2012). Outcomes of childhood pulmonary arterial hypertension in BMPR2 and ALK1 mutation carriers. Am. J. Cardiol..

[24] Miyamoto K., Inai K., Kobayashi T., Maeda J., Takatsuki S., Nakayama T., Furutani Y., Yamagishi H., Nakanishi T. (2021). Outcomes of idiopathic pulmonary arterial hypertension in Japanese children: A retrospective cohort study. Heart Vessel..

[25] Zhu N., Gonzaga-Jauregui C., Welch C.L., Ma L., Qi H., King A.K., Krishnan U., Rosenzweig E.B., Ivy D.D., Austin E.D. (2018). Exome Sequencing in Children With Pulmonary Arterial Hypertension Demonstrates Differences Compared With Adults. Circ. Genom. Precis. Med..

[26] Austin E.D., Phillips J.A., Cogan J.D., Hamid R., Yu C., Stanton K.C., Phillips C.A., Wheeler L.A., Robbins I.M., Newman J.H. (2009). Truncating and missense BMPR2 mutations differentially affect the severity of heritable pulmonary arterial hypertension. Respir. Res..

[27] Vonk Noordegraaf A., Westerhof B.E., Westerhof N. (2017). The Relationship Between the Right Ventricle and its Load in Pulmonary Hypertension. J. Am. Coll. Cardiol..

[28] Hautefort A., Mendes-Ferreira P., Sabourin J., Manaud G., Bertero T., Rucker-Martin C., Riou M., Adao R., Manoury B., Lambert M. (2019). Bmpr2 Mutant Rats Develop Pulmonary and Cardiac Characteristics of Pulmonary Arterial Hypertension. Circulation.

