# Supplementary material for: Association between Genotype, Presentation, and Outcome in Childhood Idiopathic and Hereditary Pulmonary Arterial Hypertension
Source: J Clin Med. 2022 Dec 9;11(24):7331. doi: 10.3390/jcm11247331 (PMC9783670; doi:10.3390/jcm11247331)

Figure S1. Clinical characteristics and hemodynamics in subgroups: Comparisons between patients with pathogenic and VUS in BMPR2 variants or pathogenic/likely pathogenic variants in other PAH related genes, and no pathogenic variants in age at diagnosis, WHO FC, BNP level, RAP, PVRI and cardiac index (CI). (n=62 in no variants, n=67 in BMPR2 or pathogenic/likely pathogenic variants in other PAH related genes).

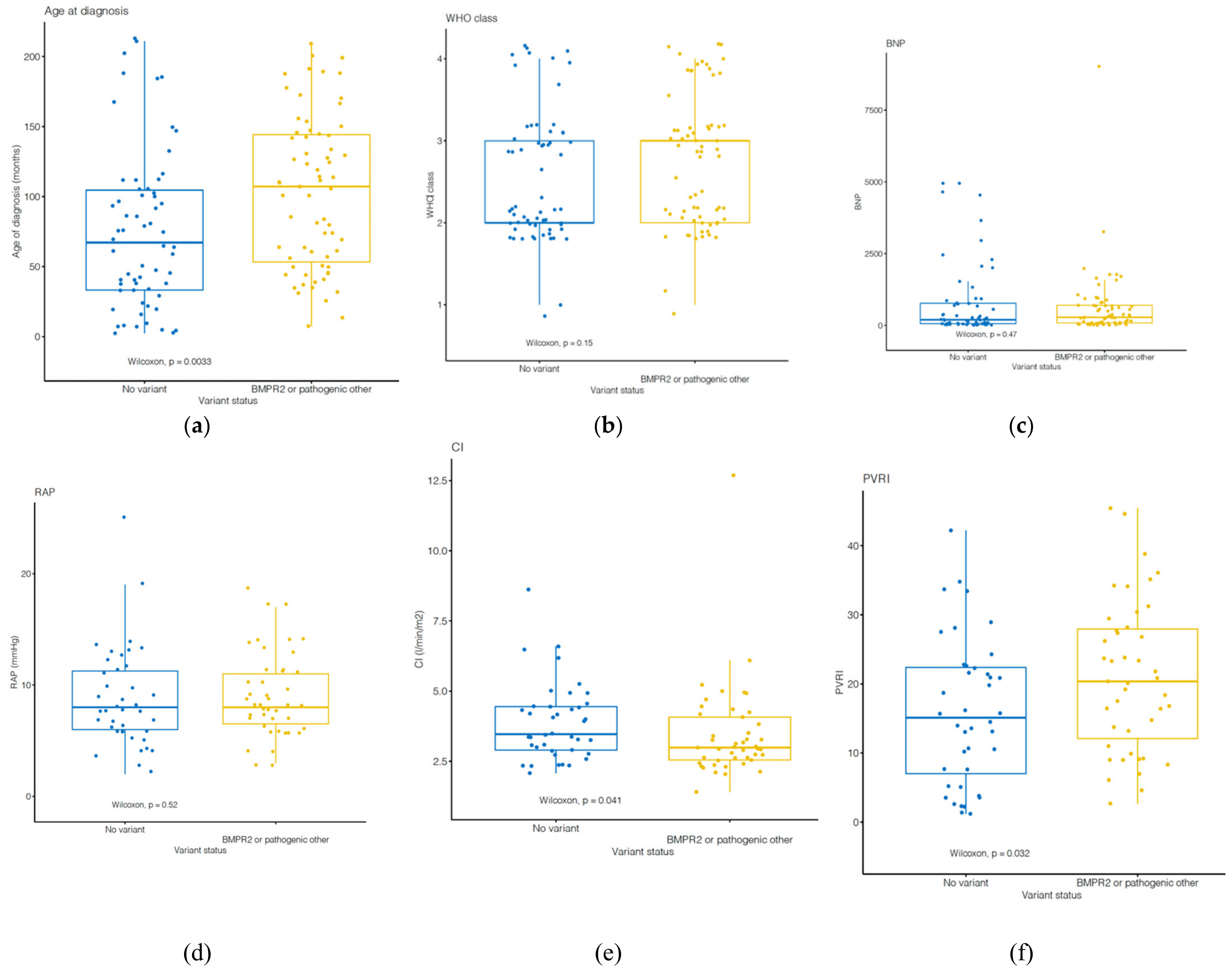

Figure S2. Kaplan-Meier survival analysis in patients with VUS BMPR2 and pathogenic BMPR2.

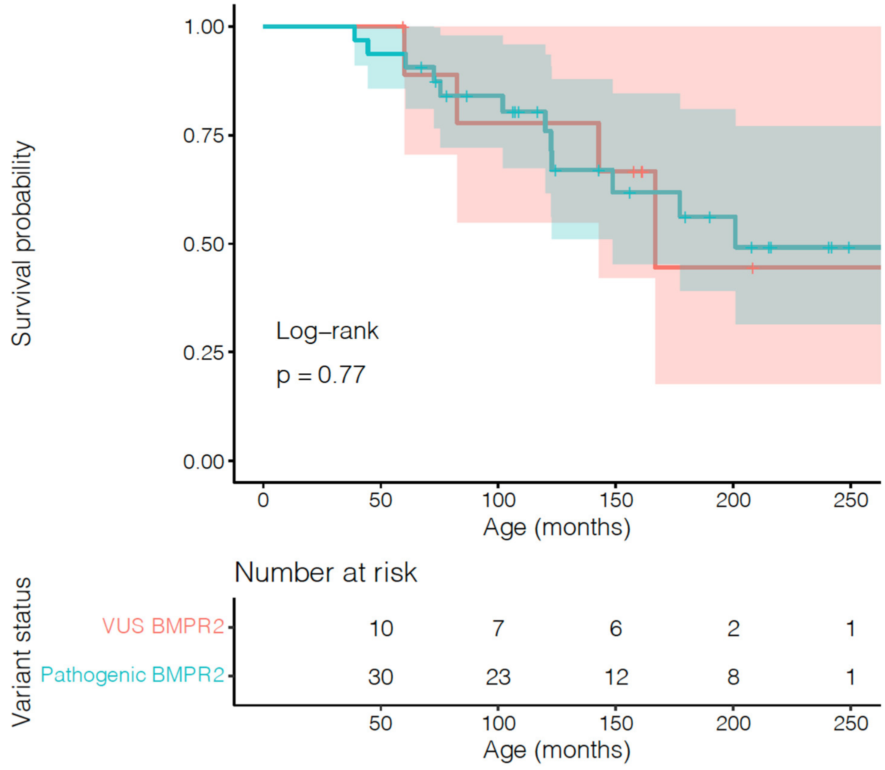

Supplement: Supplementary file 1 [file jcm-11-07331-s001.zip › jcm-2042413-supplementary.pdf]
